# Cheating Death: A Rare Case Presentation of Kratom Toxicity

**DOI:** 10.7759/cureus.16582

**Published:** 2021-07-23

**Authors:** Palak Patel, Mina Aknouk, Shawn Keating, Ivan Richard, Priyaranjan Kata, Rana Y Ali, Pramil Cheriyath

**Affiliations:** 1 Internal Medicine, Hackensack Meridian Ocean Medical Center, Brick, USA; 2 Medicine, Hackensack Meridian Ocean Medical Center, Brick, USA; 3 Pulmonology, Hackensack Meridian Ocean Medical Center, Brick, USA

**Keywords:** kratom, rhabdomyolysis, cardiomyopathy, transient nonischemic reversible cardiomyopathy, cerebrovascular accident

## Abstract

Kratom is a psychoactive herb that has been gaining widespread popularity due to its ease of availability and opiate-like effects. While it has been used beneficially as a method of weaning off opiate addictions, it does have a host of toxic effects when misused or abused. There has been a wide spectrum of negative effects including renal failure, liver failure, and cardiac toxicity. While some adverse effects have been reversed with medical intervention, others left more of a detrimental long-term impact not amenable to even the most invasive therapies. We present the case of a patient who was admitted to the intensive care unit after presenting with unresponsiveness secondary to a cerebrovascular accident, rhabdomyolysis, and renal failure. The patient had begun using kratom, initially for recreational purposes, and later escalating it to abusive doses. The patient survived the episode after suffering many complications including transient reversible nonischemic cardiomyopathy and was discharged in a neurologically stable state; however, he ended up being hemodialysis-dependent at such an early age. Rhabdomyolysis is a rare complication of this herb that has not been well documented.

## Introduction

*Mitragyna speciosa* is an indigenous southeast Asian herb known as kratom. Kratom is a psychoactive herb with both opiate and stimulant-like properties. Due to its accessibility, kratom use in the United States has been increasing at an alarming rate. The Drug Enforcement Administration classified kratom as a schedule I drug in 2016; however, the decision was withdrawn because of claims that it can help those with opioid dependency. Kratom contains 7-hydroxy mitragynine and mitragynine, which act on µ-opioid receptors and are more potent than morphine [1]. Kratom has risen in popularity as a method of withdrawal assistance. Regular kratom use can lead to adverse effects, such as tachycardia, hypertension, renal failure, hepatocellular injury, cognitive impairment, behavioral impairment, nausea, agitation, lethargy, and other symptoms similar to that of opioid withdrawal [2]. At this time, while not approved by the Food and Drug Administration, it has been documented that kratom can be reversed with naloxone reversal [3]. The estimated current prevalence in the US population is 0.8% with a mean age of 35 [4]. US poison centers received 660 calls about reported exposure to kratom [5]. Kratom is not part of the routine drug screening and hence often goes undetected; even with specialized testing, it can take up to two weeks to obtain results [6]. It is crucial to recognize the drug's presentation and be vigilant despite a negative drug screen in the appropriate clinical setting as kratom overdose can be lethal as evident from severe hepatic injury, renal failure, and cardiotoxicity that ensues [7].

## Case presentation

A 28-year-old male with a remote past medical history of alcohol and opiate abuse and questionable seizures was brought into the emergency department by emergency medical services after being found unresponsive at home by his girlfriend. The girlfriend reports last communicating with the patient approximately 16 hours prior to the presentation via text messaging. She was concerned and decided to check up on him after he did not answer her text or calls.

There was no other significant surgical history. No medical allergies were noted. Family history was reviewed and was not contributory. He was not on any anti-epileptic drugs and home medication only included pantoprazole for gastroesophageal reflux disease.

Patient received 4 mg of intranasal Narcan, 2 mg of intraosseous Narcan without any response. He was intubated in the field with an 8.0 endotracheal tube. Upon arrival at the emergency room, the patient underwent a computed tomography (CT) of the head, which did not reveal any acute intracranial bleeding or other pathology. Patient, however, was noted to have myoclonic jerking and remained unresponsive on the ventilator. Vital signs were temperature of 90.8 degrees Fahrenheit, blood pressure of 147/89 mmHg, a pulse of 114 beats per minute, respiratory rate 21 rpm, and pulse oximetry 95% on 50% FiO_2_ on the ventilator. On physical examination, the patient had intermittent myoclonic jerking, pupils were equally round, and reactive to light, normal S1, S2, regular rate and rhythm, lungs were clear to auscultation bilaterally, abdomen was soft, nondistended and extremities had no edema but left lower extremity had a linear rash anteriorly.

Admission laboratory results are summarized in Table 1. Arterial blood gas on 100% FiO_2_ revealed pH of 7.045 (7.35-7.45), pCO_2_ of 56.3 mmHg (35-45 mmHg), pO_2_ of 270.7 mmHg (75-100 mmHg), and bicarbonate of 15.1 mmol/L (22-28 mmol/L).

**Table 1:** Admission laboratory results Abbreviations: WBC, white blood cell; BUN, blood urea nitrogen; AST, aspartate aminotransferase; ALT, alanine aminotransferase; CPK, creatinine phosphokinase.

Laboratory findings	Results	Normal range
WBC	22.4 x 10^3^/uL	4.5-11.0 x 10^3^/uL
Hemoglobin	16.1 g/dL	13.2-17.5 g/dL
Platelets	211 x 10^3^/uL	140-450 x 10^3^/uL
Sodium	137 mmol/L	136-145 mmol/L
Potassium	5.9 mmol/L	3.5-5.2 mmol/L
Chloride	96 mmol/L	96-110 mmol/L
Bicarbonate	19 mmol/L	24-31 mmol/L
BUN	30 mg/dL	5-25 mg/dL
Creatinine	3.57 mg/dL	0.61-1.24 mg/dL
Glucose	116 mg/dL	70-99 mg/dL
AST	1932 U/L	10-60 U/L
ALT	126 U/L	38-126 U/L
Total bilirubin	0.7 mg/dL	0.2-1.3 mg/dL
Alkaline phosphatase	126 U/L	38-126 U/L
Lactate	10.8 mmol/L	0.5-2.0 mmol/L
Anion gap	22 mmol/L	5-13 mmol/L
CPK	3860 iU/L	22-232 iU/L
Troponin	1.73 ng/mL	<0.04 ng/mL

Urinalysis showed amber, turbid urine with large blood and 100 mg/dL protein. The urine toxicology screen was negative for amphetamines, barbiturates, benzodiazepines, cocaine, cannabinoid, and opiates. Serum acetaminophen level was <10 ug/mL (10-30 ug/mL) and serum salicylate level was 6.0 mg/dL (15-30 mg/dL). The hepatitis panel was negative. EKG revealed normal sinus rhythm and nonspecific changes without evidence of ischemic changes.

Patient was admitted to the intensive care unit for further management of ventilator-dependent respiratory failure, acute renal failure (ARF), severe metabolic acidosis, rhabdomyolysis, and shock liver. Cardiology, infectious disease, neurology, and nephrology were consulted. Initial hypothesis was presumed to be underlying seizure with postictal state resulting in hypoxemia with concerns for anoxic brain injury. Further inquiry from his girlfriend and family revealed that the patient had been abstinent from drugs for the past 10 years; however, he relapsed one week ago after his father passed away. Patient had started using kratom one week prior to presentation. Poison control was notified and updated routinely. Subsequent urine test results several days later confirmed the presence of high levels of mitragynine (kratom).

Clinical course

On admission, the patient was critically ill but hemodynamically stable. Shortly after, the patient decompensated and became hypotensive. An emergent central line was placed at the bedside for fluid and pressor administration. Unfortunately, it also resulted in an iatrogenic pneumothorax, which required bedside chest tube placement for stabilization.

Further trending of labs (as illustrated in Table 2) showed worsening renal failure and persistent hyperkalemia despite medical management in conjunction with diminishing urine output. Creatine kinase increased drastically to 26,989 iU/L (22-232 iU/L), alanine aminotransferase to 6409 U/L (10-24 U/L), and aspartate aminotransferase to 6005 U/L (10-60 U/L). An emergent temporary dialysis catheter was placed and hemodialysis was initiated for the management of ARF from acute tubular necrosis secondary to severe rhabdomyolysis, complicated with severe hyperkalemia.

**Table 2:** Pertinent labs during course of stay Abbreviations: AST, aspartate aminotransferase; ALT, alanine aminotransferase; TNI, troponin I.

Lab (normal value)	On admission	8 hours later (prompting dialysis)	1 day after emergent dialysis	5 days after emergent dialysis	On discharge (day 18)
Potassium (3.5-5.2 mmol/L)	5.9	6.6	5.8	3.2	4.9
Creatinine (0.61-1.24 mg/dL)	3.57	2.99	3.09	6.42	9.60
Creatine kinase (22-232 iU/L)	3860	-	26,989	2856	-
AST (10-60 U/L)	1932	-	6005	323	15
ALT (38-126 U/L)	2748	-	6409	1911	47
TNI (<0.04 ng/mL)	1.73	8.32	9.37	-	-

**Figure 1:**
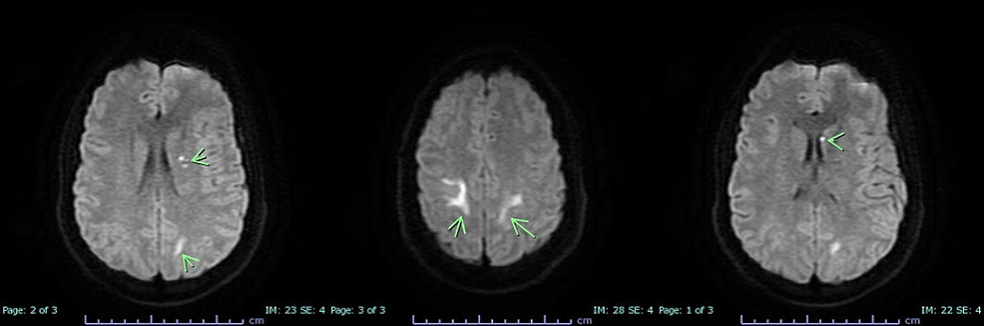
MRI of the brain showing cerebrovascular accident of posterior parietal lobes bilaterally and left basal ganglia

Troponin peaked at 9.37 ng/mL and it was deemed as demand ischemia per cardiology in the absence of ischemic changes on EKG. A transthoracic echocardiogram was done, which revealed a severely reduced left ventricular systolic function with an ejection fraction (EF) of 31-35%. Furthermore, MRI (Figure 1) of the brain was performed, which revealed multifocal acute infarcts involving the posterior parietal lobes bilaterally and left basal ganglia. He then underwent a transesophageal echocardiogram to ascertain the presence of a thrombus or mass in the left atrium. The results were unremarkable without evidence of a cardiac source of emboli. Much to the team’s surprise, it was noted that cardiac function was also restored with a noted normal EF. Normal cardiac contractility and EF were observed when the patient underwent a LexiScan nuclear stress test, which showed homogeneous distribution of radiotracer at the left ventricle on stress and rest images without evidence of reversible perfusion defect. 

Patient’s course of stay was also complicated with methicillin-resistant *Staphylococcus aureus* pneumonia for which he received intravenous fluids and antibiotics. After several sessions of dialysis, the patient's mental status improved, the chest tube was removed, and was successfully extubated without any residual neurological deficits. Renal function never returned to baseline and a permanent hemodialysis catheter was placed. He was eventually discharged to a rehabilitation program in a stable condition and subsequently returned home.

## Discussion

Cardiomyopathy is a broad definition of diseases of the myocardium associated with cardiac dysfunction. The main categories of cardiomyopathy are as follows: dilated, hypertrophic, restrictive, arrhythmogenic right ventricular, and unclassified. Dilated cardiomyopathy, where the heart becomes enlarged causing a decrease in cardiac output, has numerous causes. Some of those causes include infections, genetics, alcohol, cocaine, and toxins. Dilated cardiomyopathy can lead to heart failure, committing a patient to a lifetime of impaired cardiac function. There have been cases of documented reversible cardiomyopathy: those caused by medications such as ibrutinib and amphotericin B, as well as those caused by toxins such as methamphetamine [8-10]. Our patient had transient nonischemic reversible cardiomyopathy due to the toxicity of kratom. Very few cases of such reversible nonischemic cardiomyopathy have been documented at this point due to kratom overdose [1].

Rhabdomyolysis is a potentially life-threatening condition caused by the breakdown of muscle fibers leading to muscle contents filling the circulatory system. Common causes are crush injuries, heat exhaustion, medication-induced including statins, and toxin-induced including illicit substances such as heroin. The outcomes of rhabdomyolysis can be ARF, disseminated intravascular coagulation, hepatic failure, and cardiac myopathy. Rapid recognition and treatment are necessary when dealing with these rare cases. There have been few documented cases of kratom inducing rhabdomyolysis; and upon literature review, no cases were found with patients having rhabdomyolysis, cerebrovascular accident (CVA), and transient nonischemic reversible cardiomyopathy [1,2]. 

It was believed by the patient that because kratom was purchased over the counter at a local convenience store that it would not be harmful. Besides being readily available both online and in local stores, kratom does not have a researched proven dosing amount or schedule. The opioid effect of the drug makes the risk of dependence and potential overdose a higher risk than should be allowed. The popularity of this drug has been increasing in the United States over the last decade, and with that rise has come to an exponential growth in overdose cases. Regulation will be the only way to prevent further abuse and harm from kratom [5].

## Conclusions

A review of the literature demonstrates an emerging public health threat in the United States due to the severity of reported symptoms and the sequelae of kratom use. Kratom use has been increasing for the past decade due to the ease of its availability and the popular misconception of its safety. Following the recent opioid epidemic, many are looking for alternative safe, easily available treatments, which lead them to kratom, an unregulated substance. Its opioid and stimulant properties make it an option for treating opioid withdrawal, but it comes with a great propensity for abuse. The current literature is lacking substantial research into the effects of kratom. Kratom overdose leading to rhabdomyolysis, CVA, and transient nonischemic reversible cardiomyopathy, to our knowledge, has not been described before. The mechanism of these effects is uncertain, and more research is required. The healthcare community should be aware of the wide array of adverse effects kratom can produce. In the interest of public health, we vehemently support the regulation of kratom as a schedule 1 controlled substance and the much-needed in-depth research on kratom.

## References

[1] Sangani V, Sunnoqrot N, Gargis K, Ranabhotu A, Mubasher A, Pokal M (2021). Unusual presentation of kratom overdose with rhabdomyolysis, transient hearing loss, and heart failure. J Investig Med High Impact Case Rep.

[2] Patel T, Karle E, Krvavac A (2020). Kratom: an unusual cause of rhabdomyolysis and cholestasis. Crit Care Med.

[3] Overbeek DL, Abraham J, Munzer BW (2019). Kratom (mitragynine) ingestion requiring naloxone reversal. Clin Pract Cases Emerg Med.

[4] Schimmel J, Amioka E, Rockhill K, Haynes CM, Black JC, Dart RC, Iwanicki JL (2021). Prevalence and description of kratom (Mitragyna speciosa) use in the United States: a cross-sectional study. Addiction.

[5] Anwar M, Law R, Schier J (2016). Notes from the field: kratom (Mitragyna speciosa) exposures reported to poison centers - United States, 2010-2015. MMWR Morb Mortal Wkly Rep.

[6] Sethi R, Hoang N, Ravishankar DA, McCracken M, Manzardo AM (2020). Kratom (Mitragyna speciosa): friend or foe?. Prim Care Companion CNS Disord.

[7] Diep J, Chin DT, Gupta S, Syed F, Xiong M, Cheng J (2018). Kratom, an emerging drug of abuse: a case report of overdose and management of withdrawal. A A Pract.

[8] Bandeira AC, Filho JM, de Almeida Ramos K (2016). Reversible cardiomyopathy secondary to Amphotericin-B. Med Mycol Case Rep.

[9] Liang SH, Chiu CF, Bai LY (2019). Ibrutinib-associated reversible cardiomyopathy. J Oncol Pract.

[10] Yew KL, Go CS, Razali F, Rajendran P, Ooi PS, Anum A (2014). Methamphetamine-associated reversible cardiomyopathy and stroke risk. Eur Rev Med Pharmacol Sci.

